# Supplementary material for: Direct visual observation of pedal motion-dependent flexibility of single covalent organic frameworks
Source: Nat Commun. 2023 Aug 21;14:5061. doi: 10.1038/s41467-023-40831-8 (PMC10442449; doi:10.1038/s41467-023-40831-8)
Supplement: Supplementary file 1 — Supplementary Information [file 41467_2023_40831_MOESM1_ESM.pdf]

Supplementary information

Direct visual observation of pedal motion-dependent flexibility of single covalent organic frameworks

Hongbin Chi [a], Yang Liu [a, b], Ziyi Li[a], Wanxin Chen[a], Yi He[*a]

a School of Nuclear Science & Technology, Southwest University of Science and Technology, Mianyang 621010, P. R. China.

b Sichuan College of Architectural Technology, Deyang 618000, Sichuan, P. R. China.

*Corresponding author: Prof. Dr. Yi He, Tel: +86-816-6089885, Fax: +86-816-6089889, Email: yhe2014@126.com.

Roundness

The roundness (R) can be described by

$$R = \frac{4S}{\pi L^2} \qquad (1)$$

where S and L are the area and major axis of COF-300 or COF-300-AR particles. R is directly determined by ImageJ software.

Crystal structure modeling of COF-300 and COF-300-AR

The crystal structures of COF-300 and COF-300-AR are built by Material Studio chemical structure modeling software employing the Crystal Building module. The fabricated crystal structures are geometrically optimized with the Forcite algorithm in Material Studio using the universal force field. The van der Waals and electrostatic interaction are atom based. The charges of each crystal are “use current”. The optimization quality is set as ultra-fine. The optimized minimum-energy structures are applied to simulate the powder X-ray diffraction (XRD) patterns, obtaining the simulated patterns by taking advantage of Reflex Powder Diffraction module in Material Studio. Finally, the cell parameters, space group, bond length, bond angle, vertex, and edges position are acquired by the final crystal structures.

Computational details

For calculating the energy barrier during the deformation process of COF-300, the grand canonical Monte Carlo is first used to describe the interaction between COF-300 and $CHCl_3$, and geometries are optimized[1, 2]. This calculation is implemented in the Sorption module of Materials Studio 7.0 software by performing a series of fixed-loading simulations. The Metropolis algorithm and Universal force field are selected. Other simulation parameters such as quality, charges, electrostatic, vander Waals, Ewald accuracy, and cutoff distance are set as follows: medium, use current, Ewald&Group, atom based, 0.001 kacl/mol, and 12.5 Å, respectively. After that, further geometry optimizations are conducted before and after loading different amounts of $CHCl_3$ via density functional theory. Finally, various energies such as the deformation energy (E_{def}), interaction energy (E_{int}), and total energy (E_{tot}) are calculated by using Materials Studio 7.0 software.

To investigate the pedal motion of N, 1-diphenylmethanimine, all the geometry optimizations are carried out at the MP2/cc-pVDZ level. Energy profiles along the reaction coordinate of the pedalo-type motion are performed via rigid scan, and single-point energy calculations are carried out at the CCSD(T)/cc-pVDZ level. The energy minima and transition state structures are searched, and the corresponding infrared absorption spectra are calculated to extract the characteristic vibrational frequency of C=N bonds. The Gaussian 16 software has been employed for carrying out all quantum mechanical calculations.

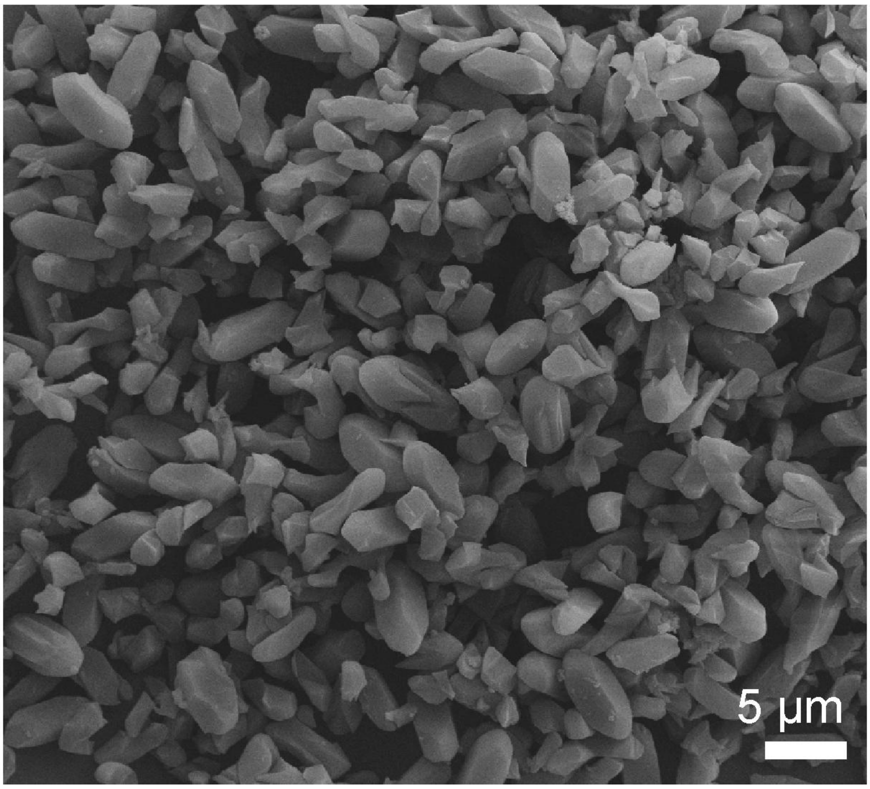

Supplementary Figure 1. SEM image of COF-300 crystals.

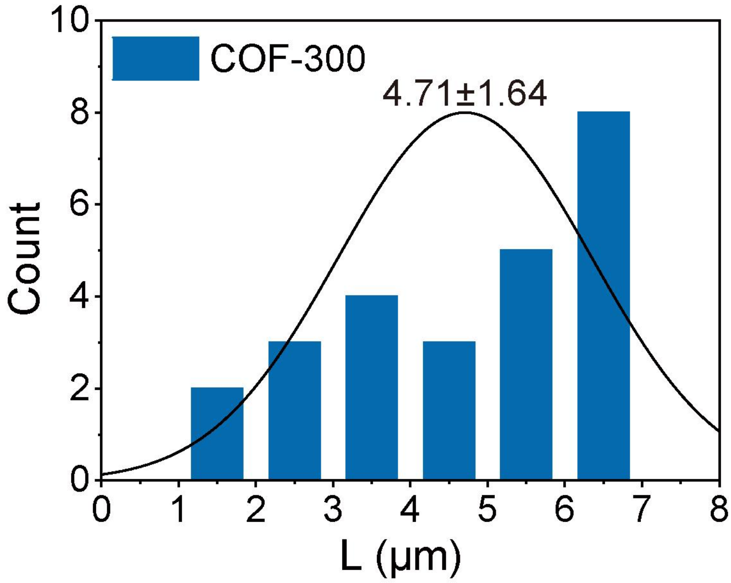

Supplementary Figure 2. Particle size (length (L)) distribution of COF-300 crystals.

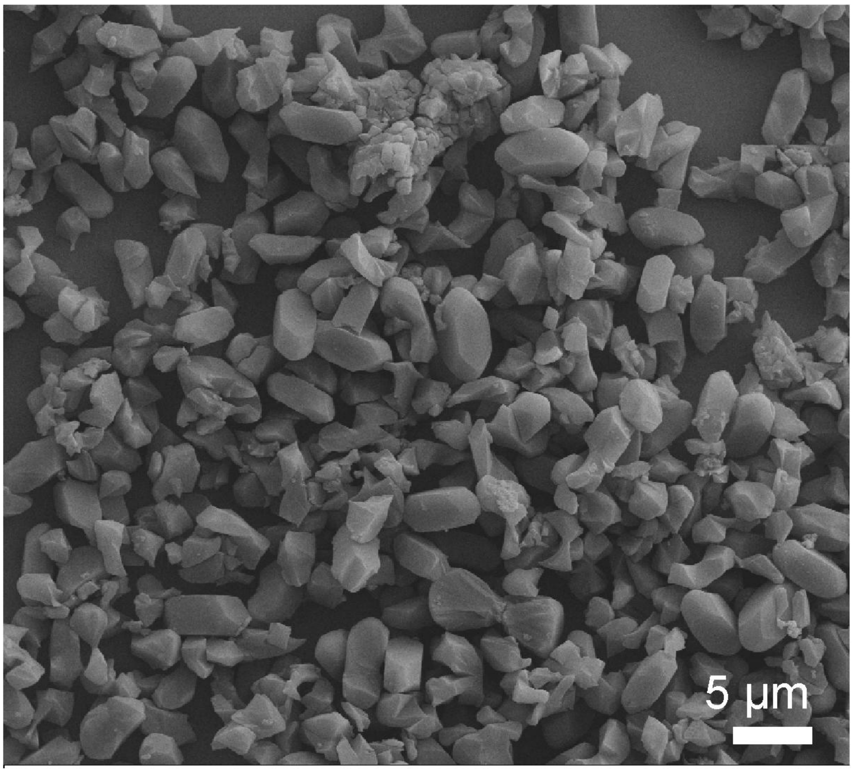

Supplementary Figure 3. SEM image of COF-300-AR crystals.

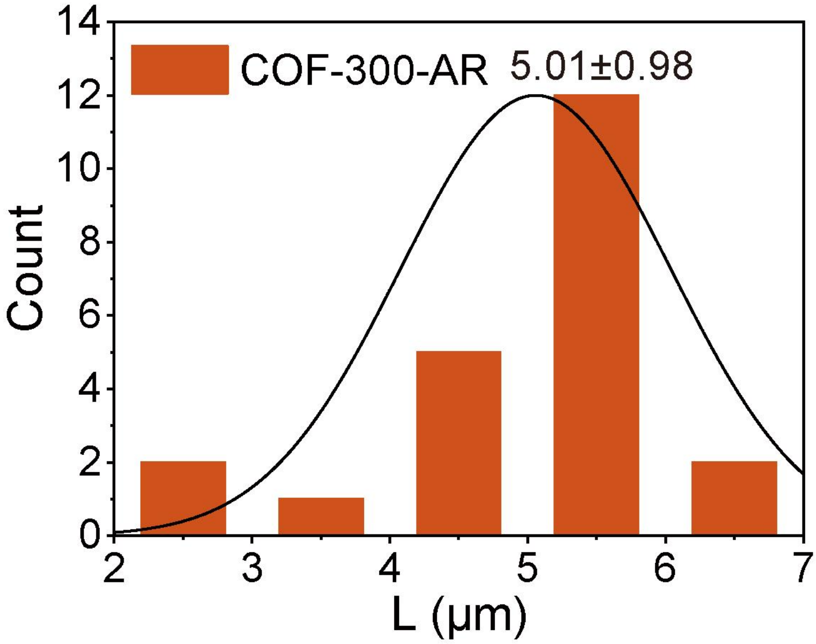

Supplementary Figure 4. Particle size (length (L)) distribution of COF-300-AR crystals.

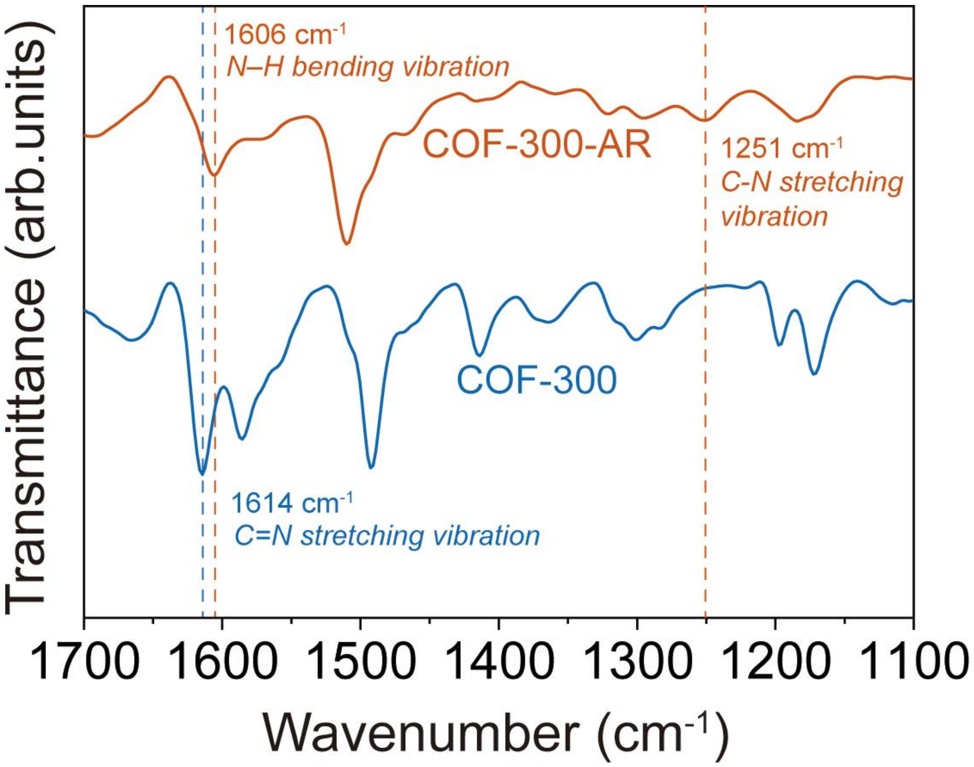

Supplementary Figure 5. Fourier transform infrared absorption spectra of COF-300 and COF-300-AR crystals.

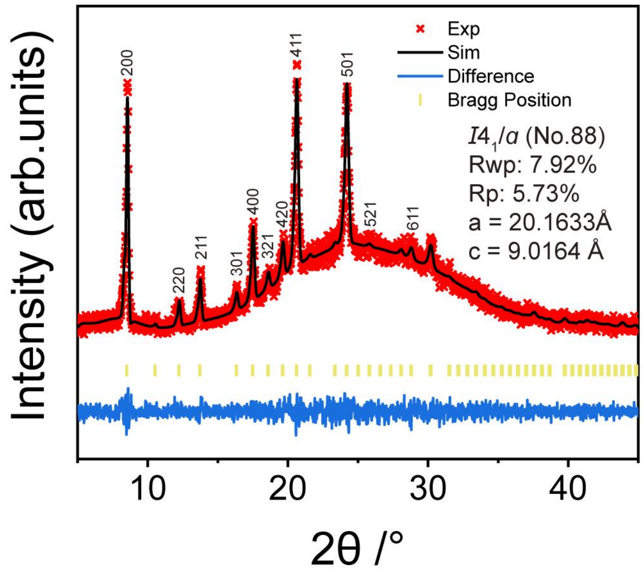

Supplementary Figure 6. XRD pattern of COF-300 crystals (red), Pawley refined (black), and difference plot (blue).

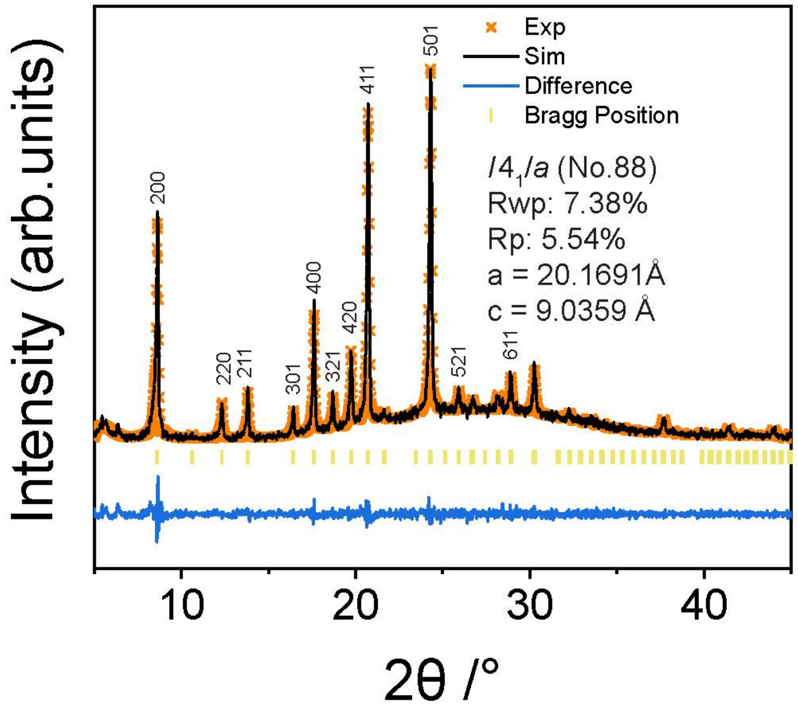

Supplementary Figure 7. XRD pattern of COF-300-AR crystals (orange), Pawley refined (black), and difference plot (blue).

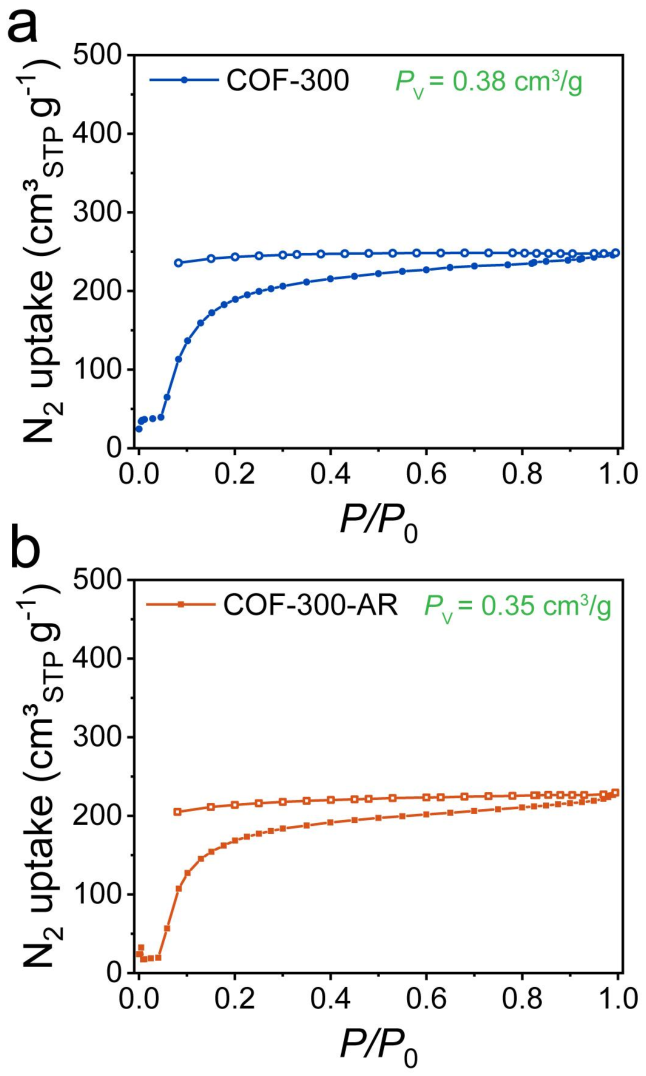

Supplementary Figure 8. N_2 adsorption-desorption measurements of (a) COF-300 and (b) COF-300-AR.

The corresponding Brunauer-Emmett-Teller (BET) surface areas are also calculated to be 1261.6 m^2/g and 886.4 m^2/g for COF-300 and COF-300-AR (Supplementary Figure 8), respectively. The decrease in the surface area of COF-300-AR can be attributed to an increase in framework mass or the generation of amorphous oligomers presented in pores after reduction[3]. Note that the desorption curve does not overlap well with the sorption process. The main reason is that the flexibility of COFs results in pore contraction, preventing the desorption of nitrogen.

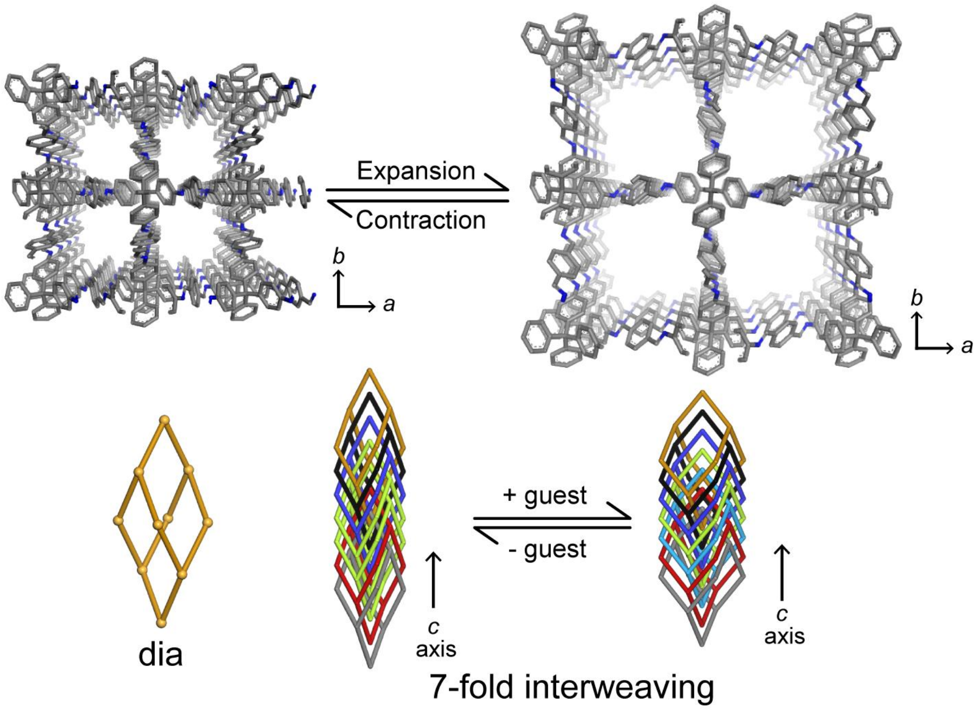

Supplementary Figure 9. Schematic diagram of the contraction and expansion of COF-300 with 7-fold interweaving upon adsorption and desorption of guest molecules.

As illustrated in Supplementary Figure 9, COF-300 has a 7-fold interpenetrated diamond topology in the tetragonal system and one-dimensional (1D) straight channels along the crystallographic c-axis[4]. Upon the adsorption of guest molecules, the adamantane-like cages of COF-300 uniformly change themselves along a- and b-axes to yield an expanded structure (Supplementary Figure 9). The 1D channels along the crystallographic c-axis can accommodate large quantities of guest molecules, whereas other directions are not accessible to the guest molecules, leading to an anisotropic deformation.

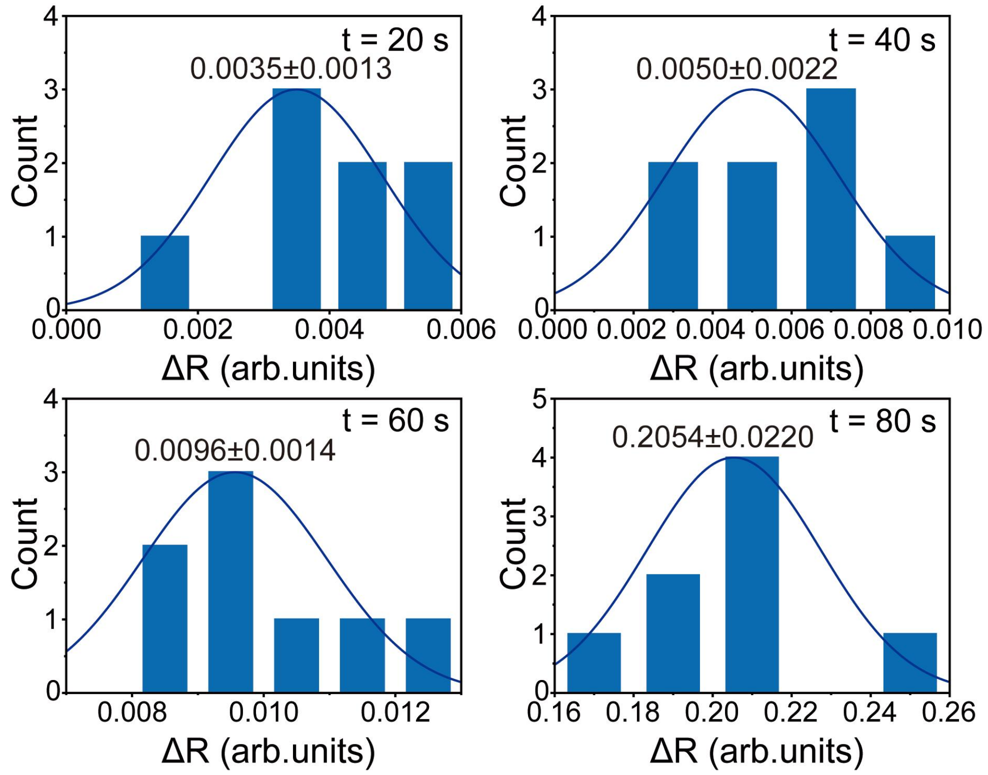

Supplementary Figure 10. Histogram distributions of COF-300 crystals in the presence of chloroform vapour (0.84 bar) under different reaction time.

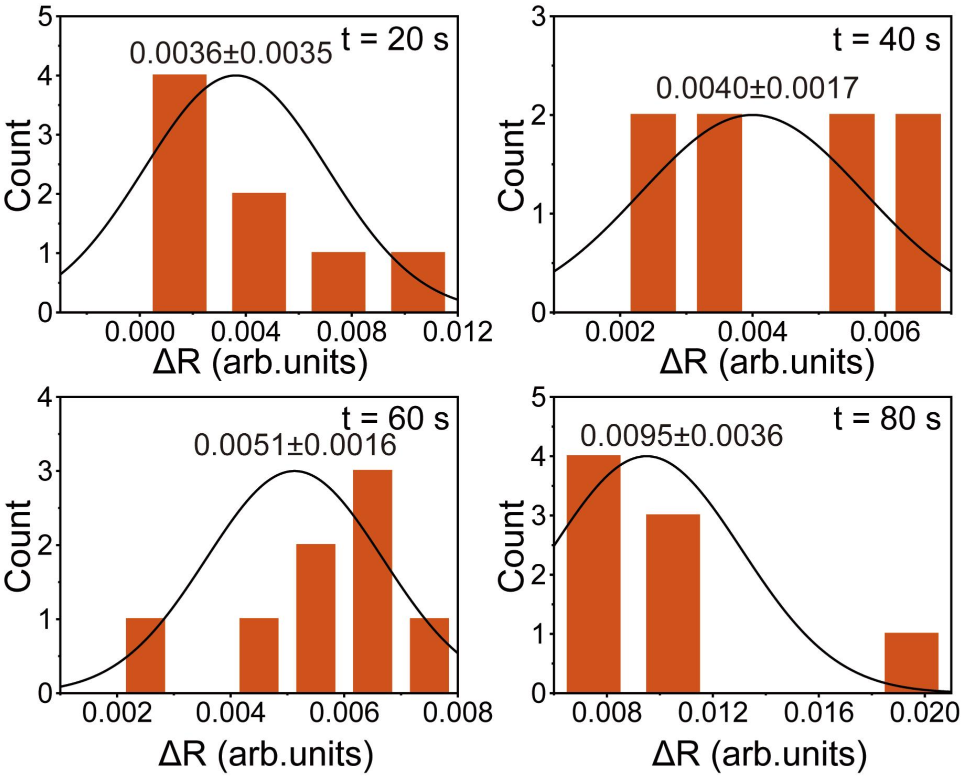

Supplementary Figure 11. Histogram distributions of COF-300-AR crystals in the presence of chloroform vapour (0.84 bar) under different reaction time.

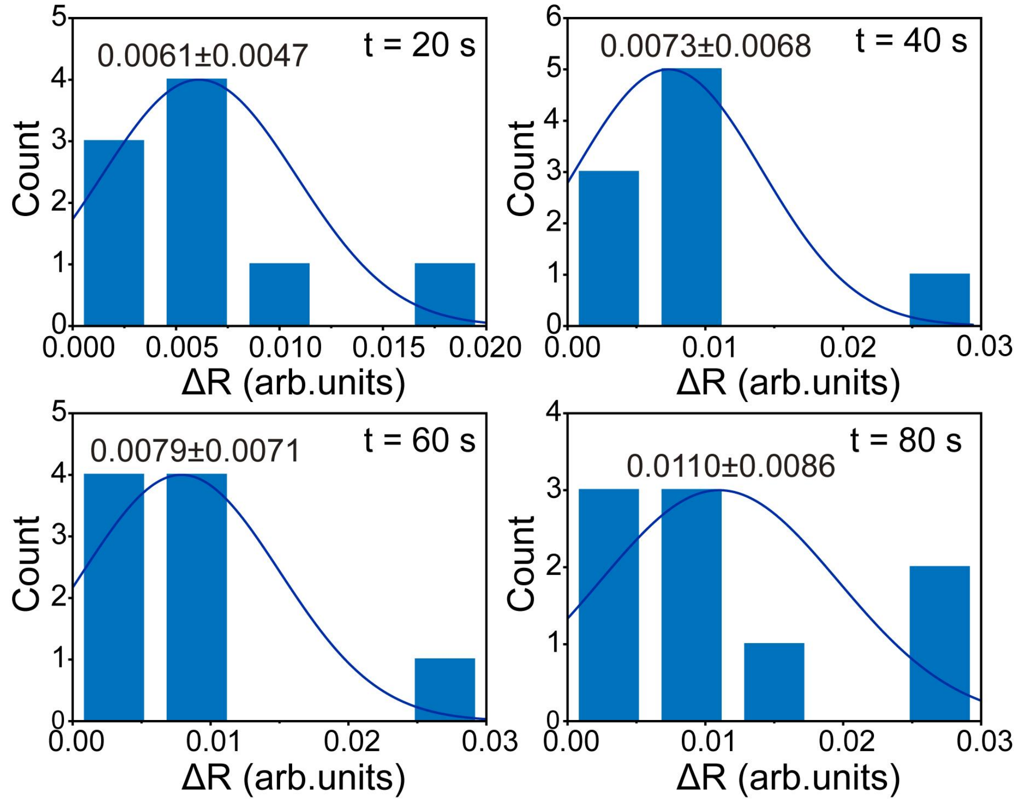

Supplementary Figure 12. Histogram distributions of COF-300 crystals in the presence of chloroform vapour (0.79 bar) under different reaction time.

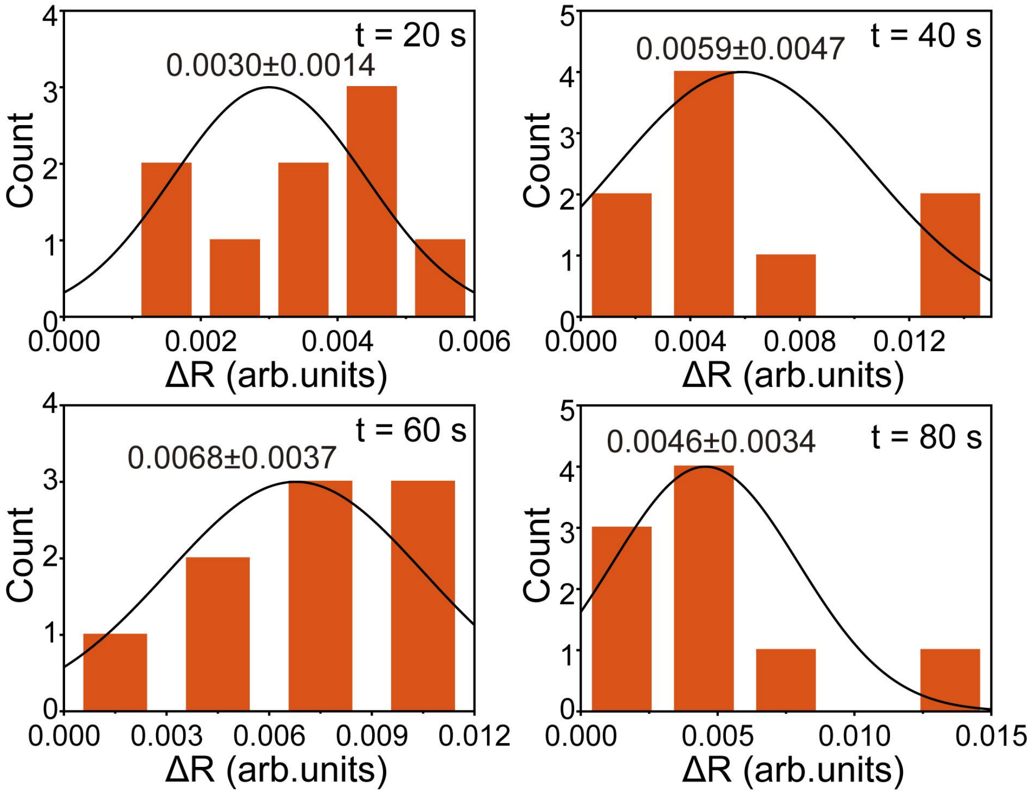

Supplementary Figure 13. Histogram distributions of COF-300-AR crystals in the presence of chloroform vapour (0.79 bar) under different reaction time.

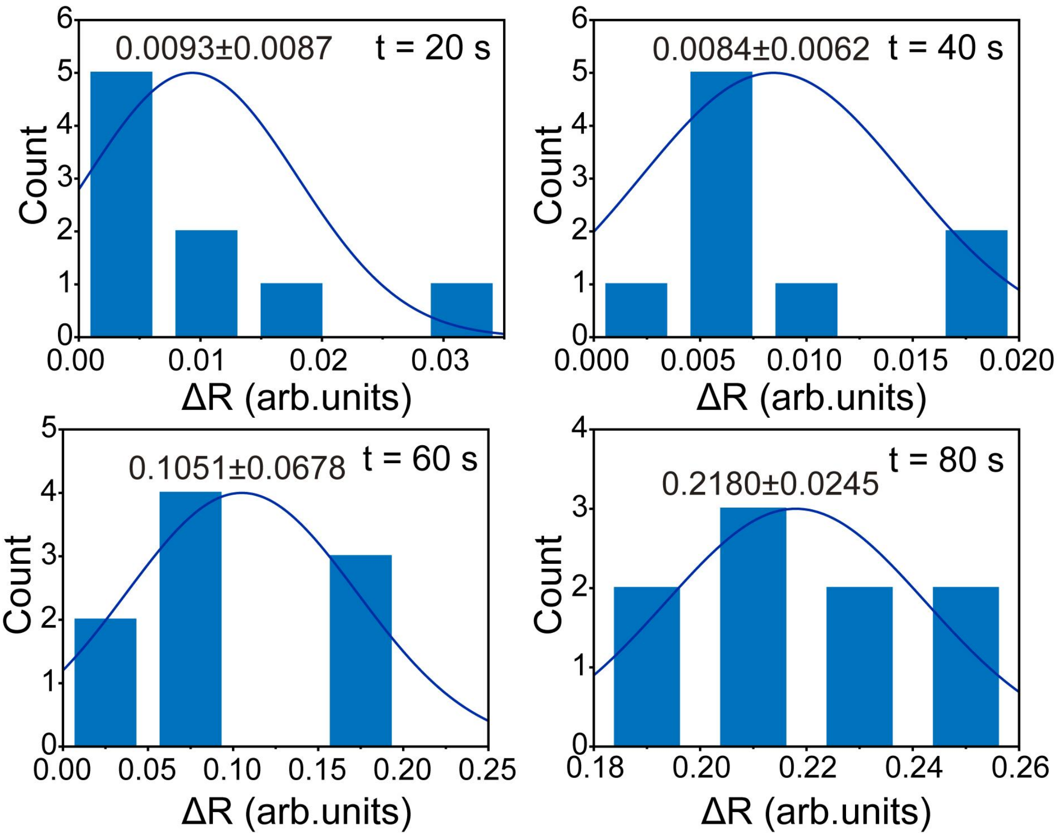

Supplementary Figure 14. Histogram distributions of COF-300 crystals in the presence of chloroform vapour (1.01 bar) under different reaction time.

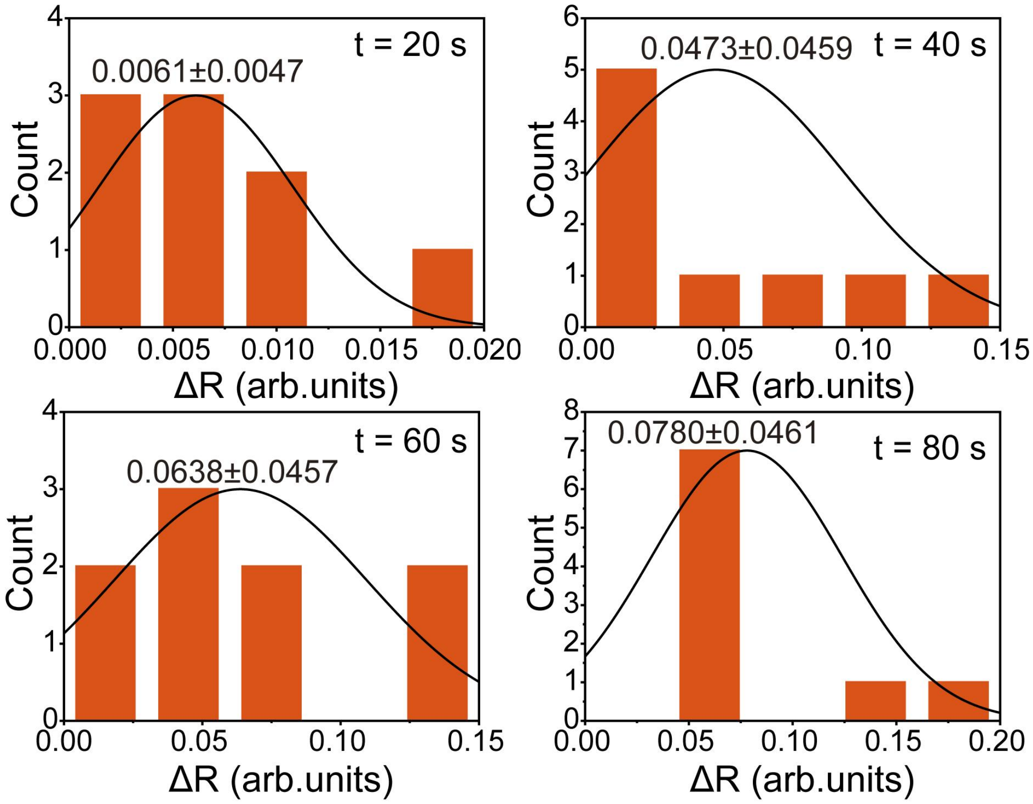

Supplementary Figure 15. Histogram distributions of COF-300-AR crystals in the presence of chloroform vapour (1.01 bar) under different reaction time.

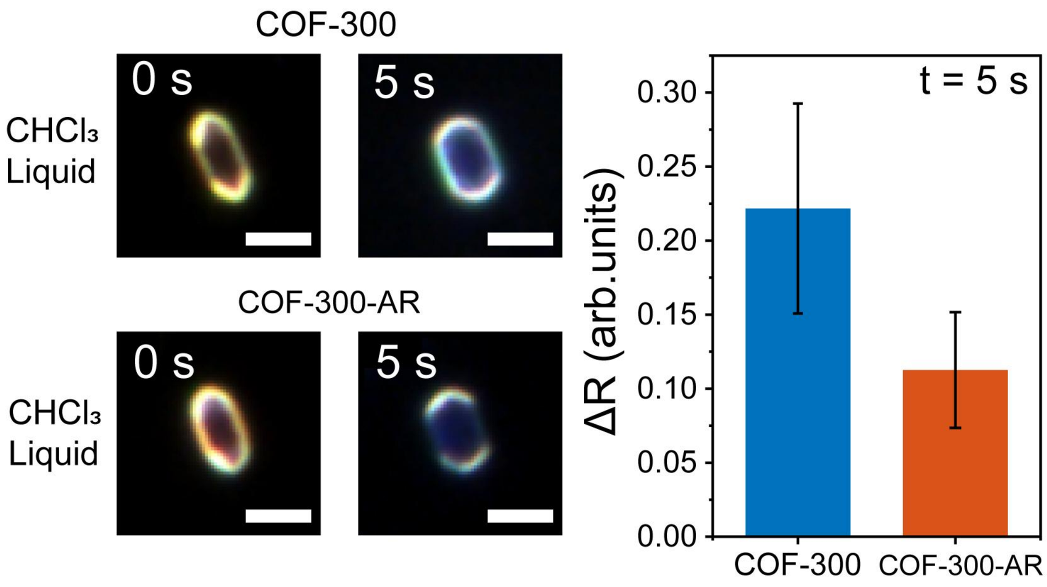

Supplementary Figure 16. DFM images of COF-300 and COF-300-AR before and after the addition of chloroform liquid, and the corresponding △R values at 5 s. Scale bars, 4 μm.

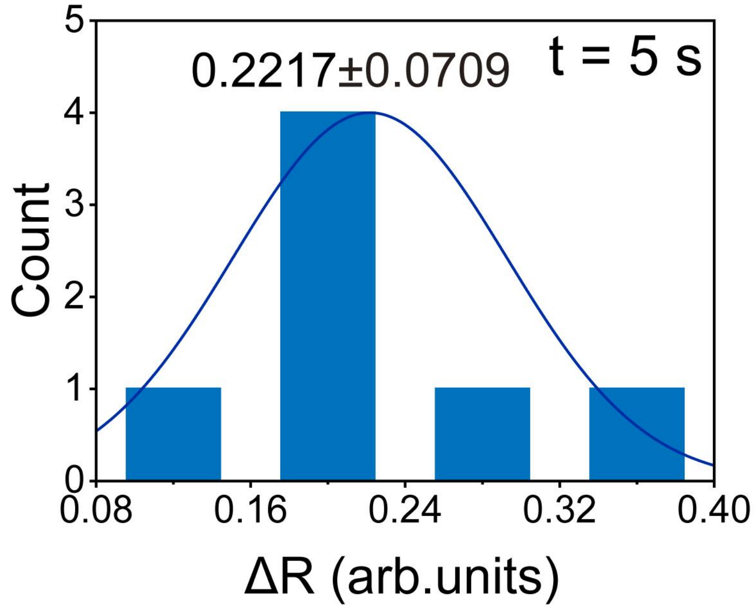

Supplementary Figure 17. Histogram distributions of COF-300 crystals in the presence of chloroform liquid.

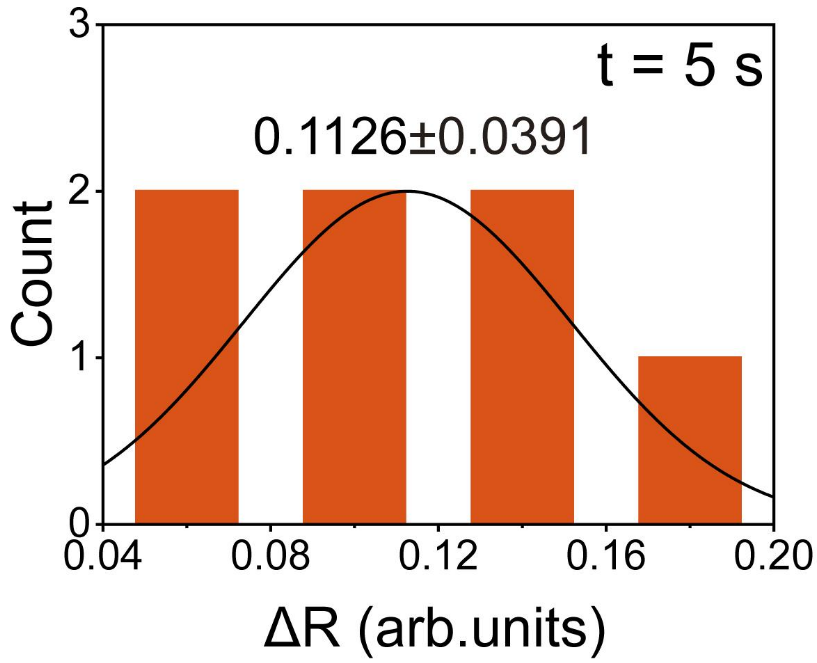

Supplementary Figure 18. Histogram distributions of COF-300-AR crystals in the presence of chloroform liquid.

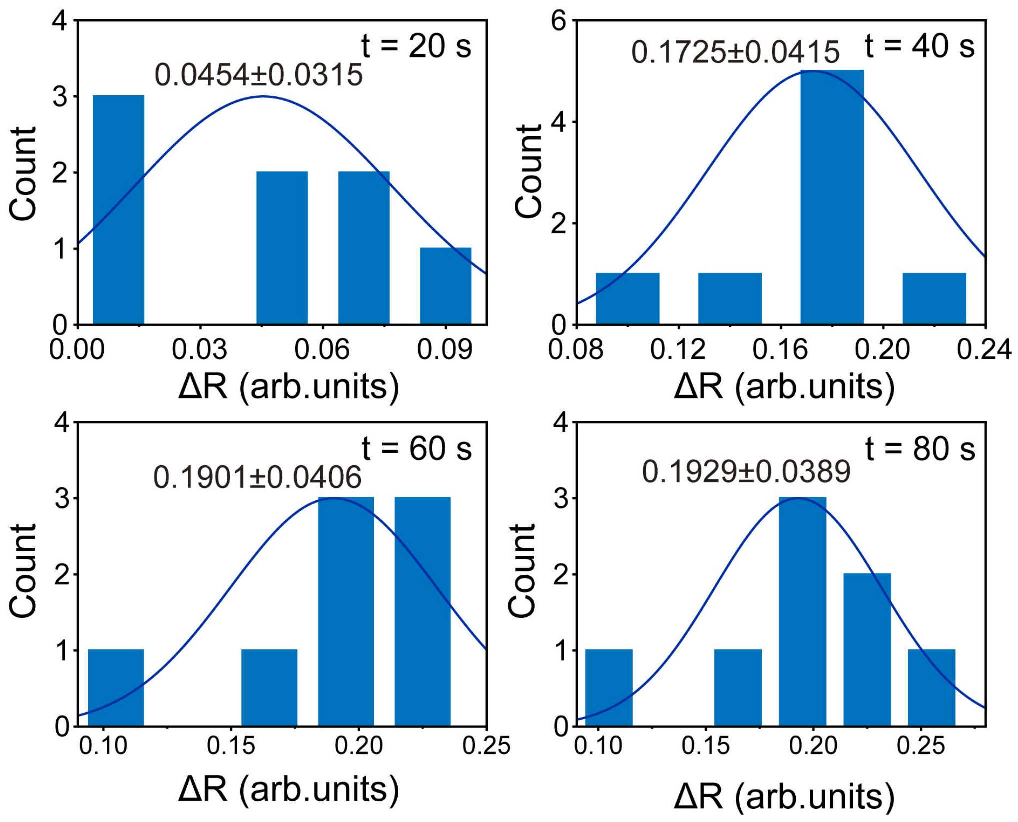

Supplementary Figure 19. Histogram distributions of COF-300 crystals in the presence of IPA vapour (0.84 bar) under different reaction time.

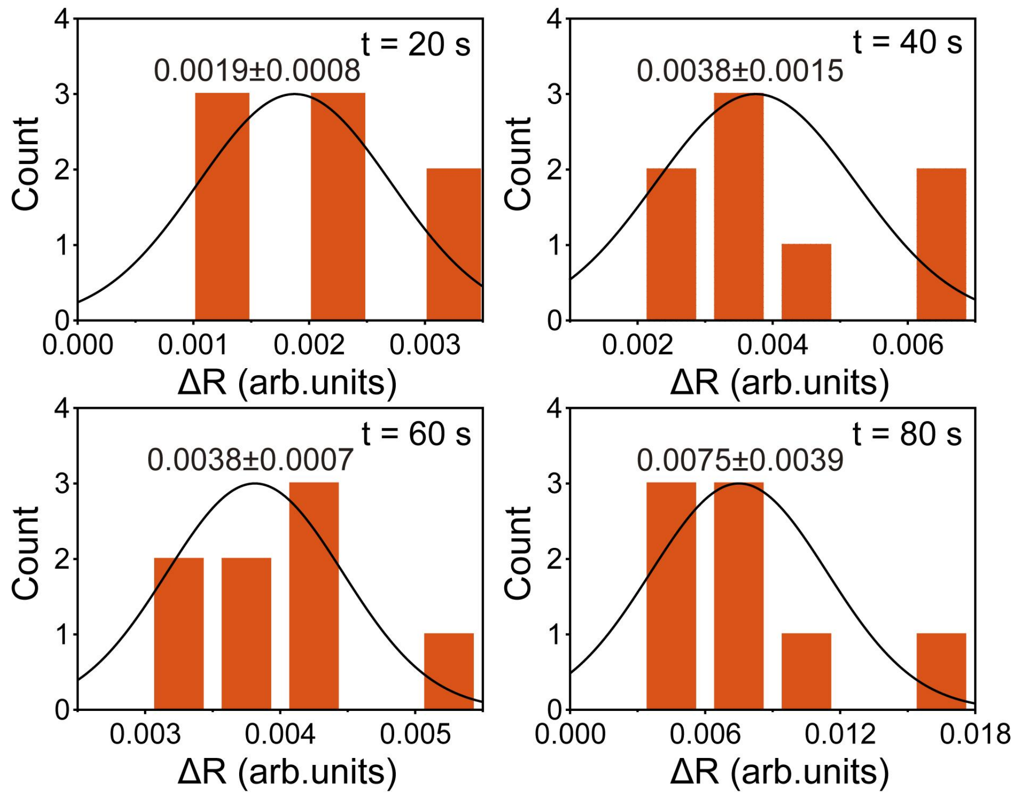

Supplementary Figure 20. Histogram distributions of COF-300-AR crystals in the presence of IPA vapour (0.84 bar) under different reaction time.

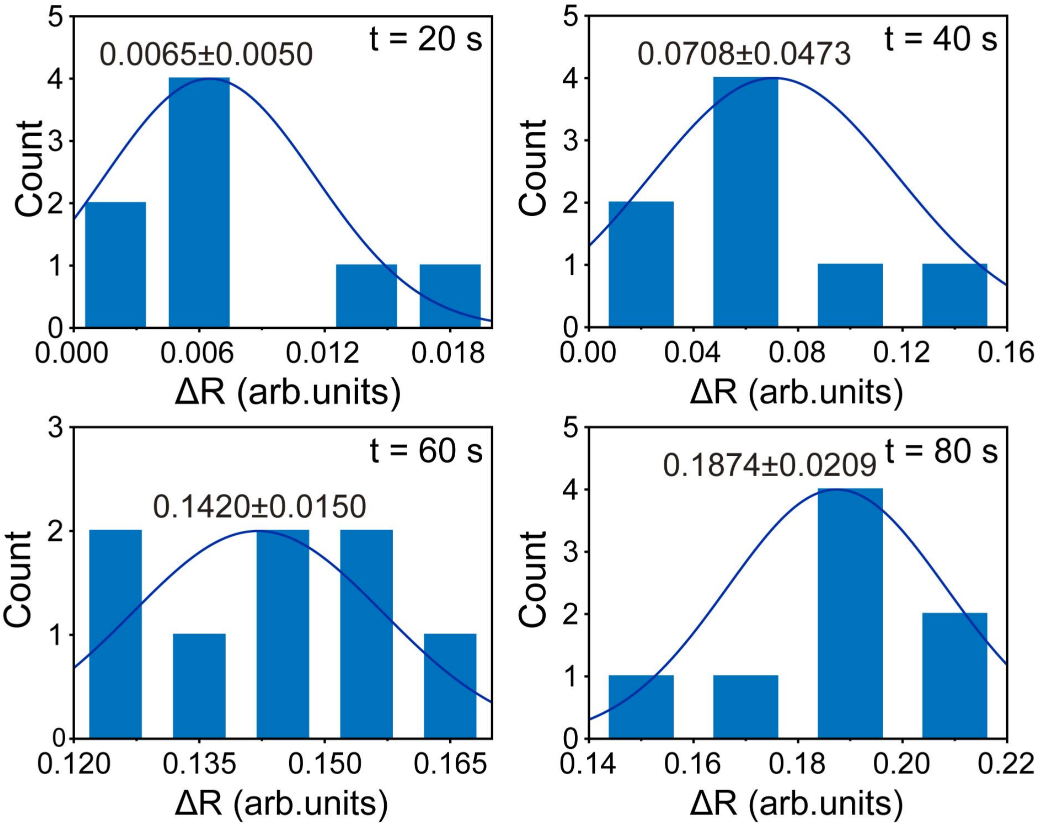

Supplementary Figure 21. Histogram distributions of COF-300 crystals in the presence of n-hexane vapour (0.81 bar) under different reaction time.

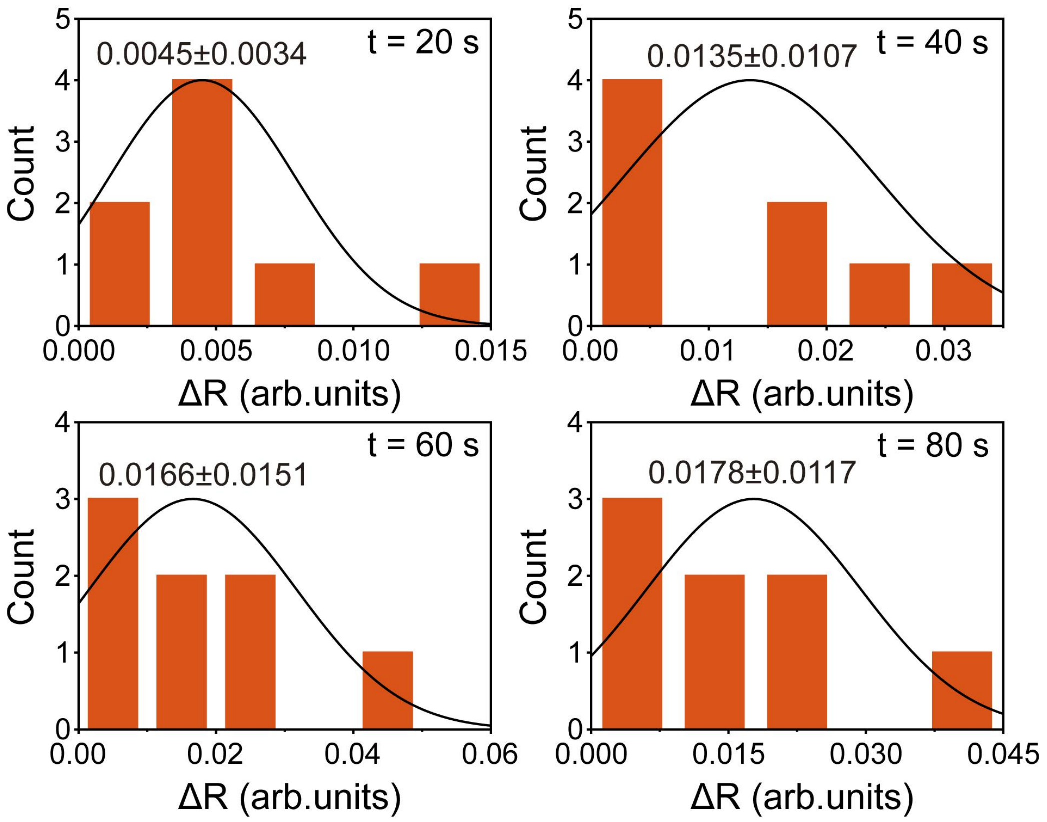

Supplementary Figure 22. Histogram distributions of COF-300-AR crystals in the presence of n-hexane vapour (0.81 bar) under different reaction time.

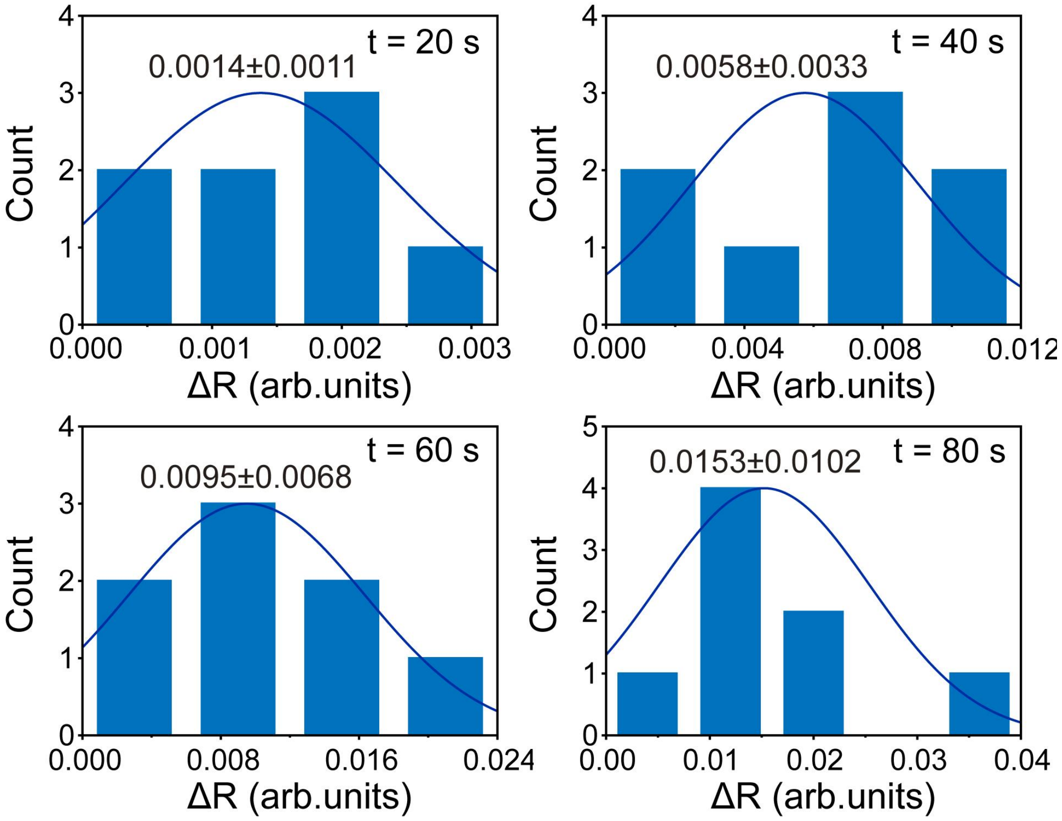

Supplementary Figure 23. Histogram distributions of COF-300 crystals in the presence of water vapour (0.69 bar) under different reaction time.

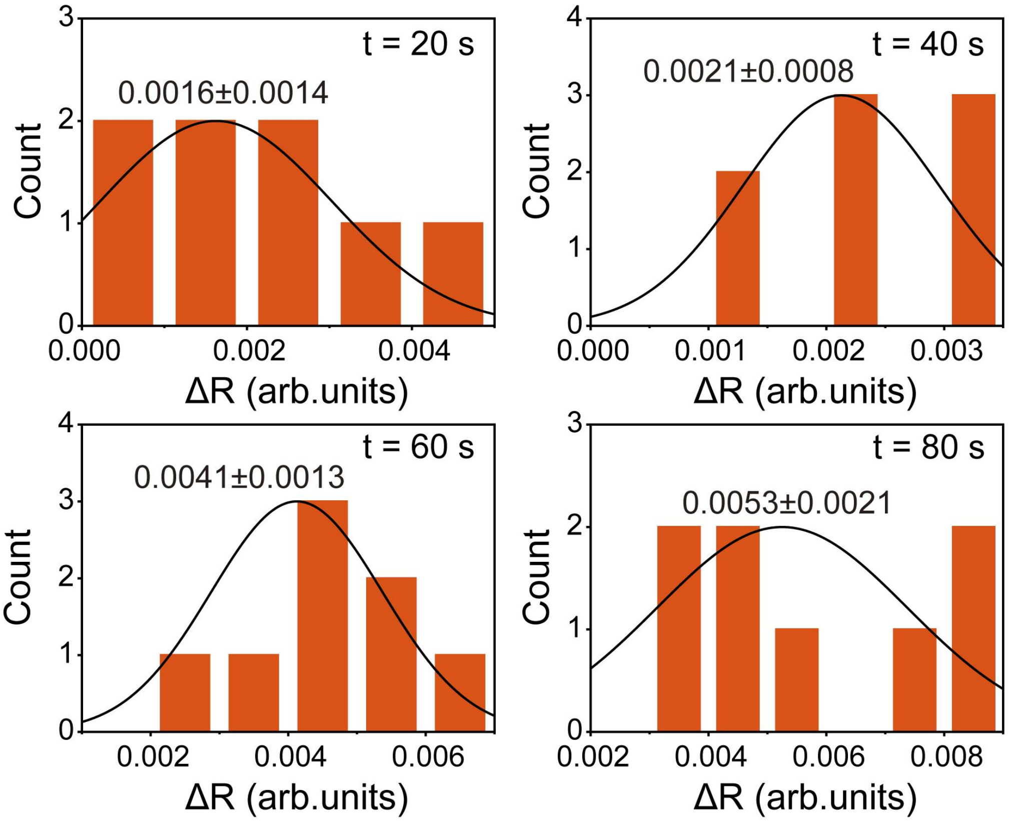

Supplementary Figure 24. Histogram distributions of COF-300-AR crystals in the presence of water vapour (0.69 bar) under different reaction time.

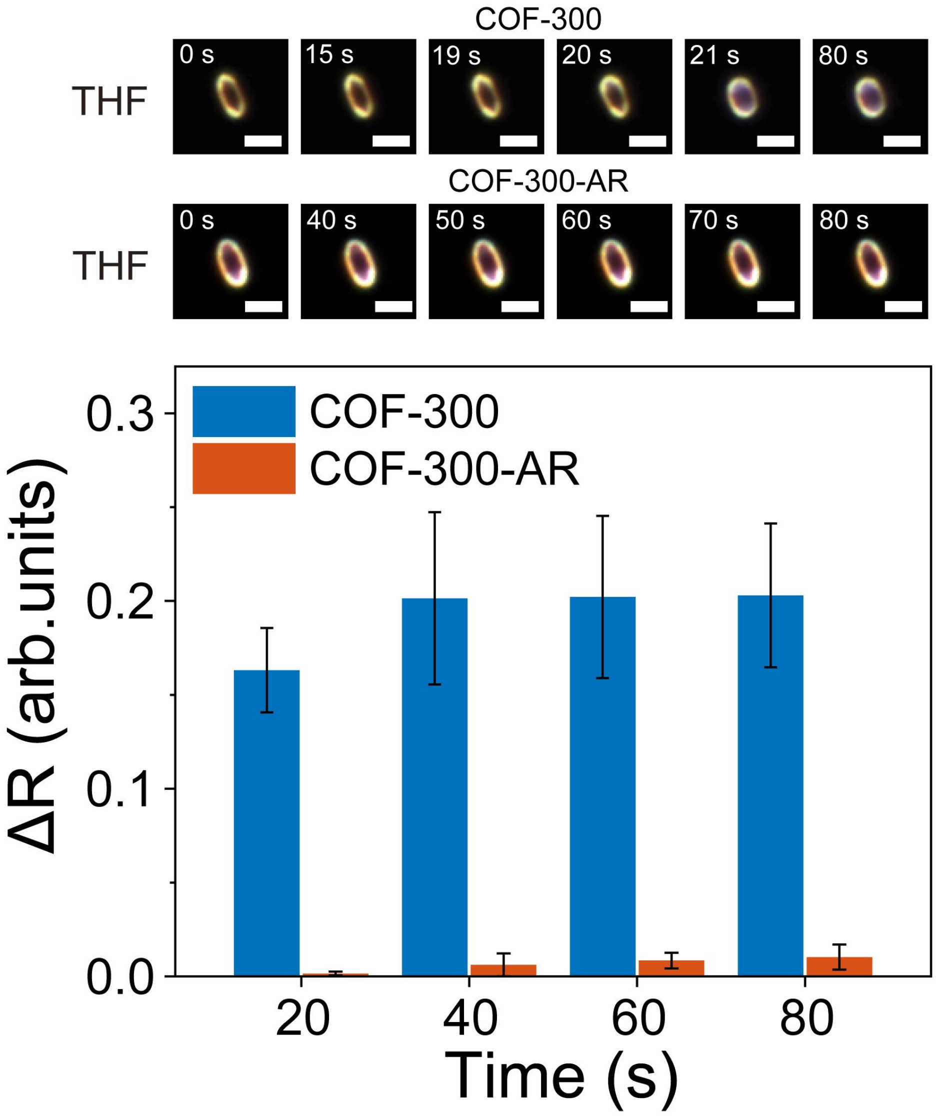

Supplementary Figure 25. Time-lapsed DFM images of single COF-300 and COF-300-AR in the presence of THF (0.88 bar) and the average roundness change (△R) of COF-300 and COF-300-AR crystals under different reaction time. Scale bars are 4 μm. Error bars stand for the standard deviations of △R.

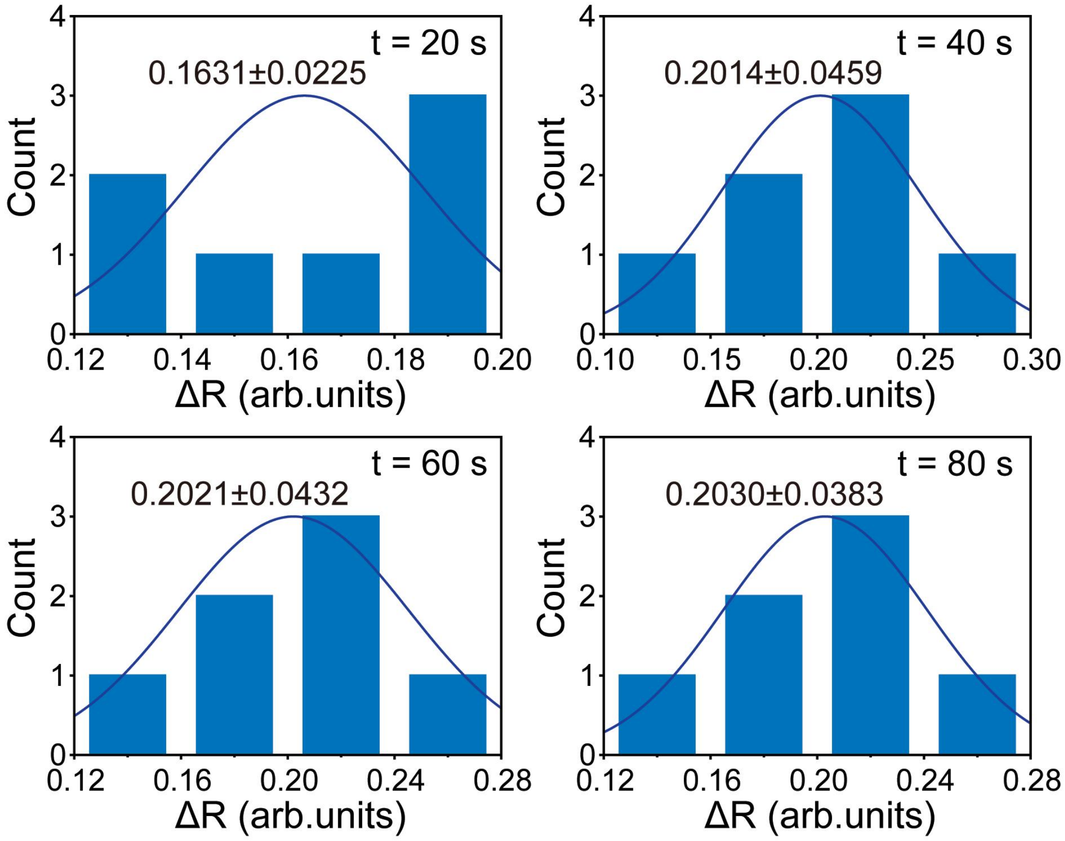

Supplementary Figure 26. Histogram distributions of COF-300 crystals in the presence of THF (0.88 bar) under different reaction time.

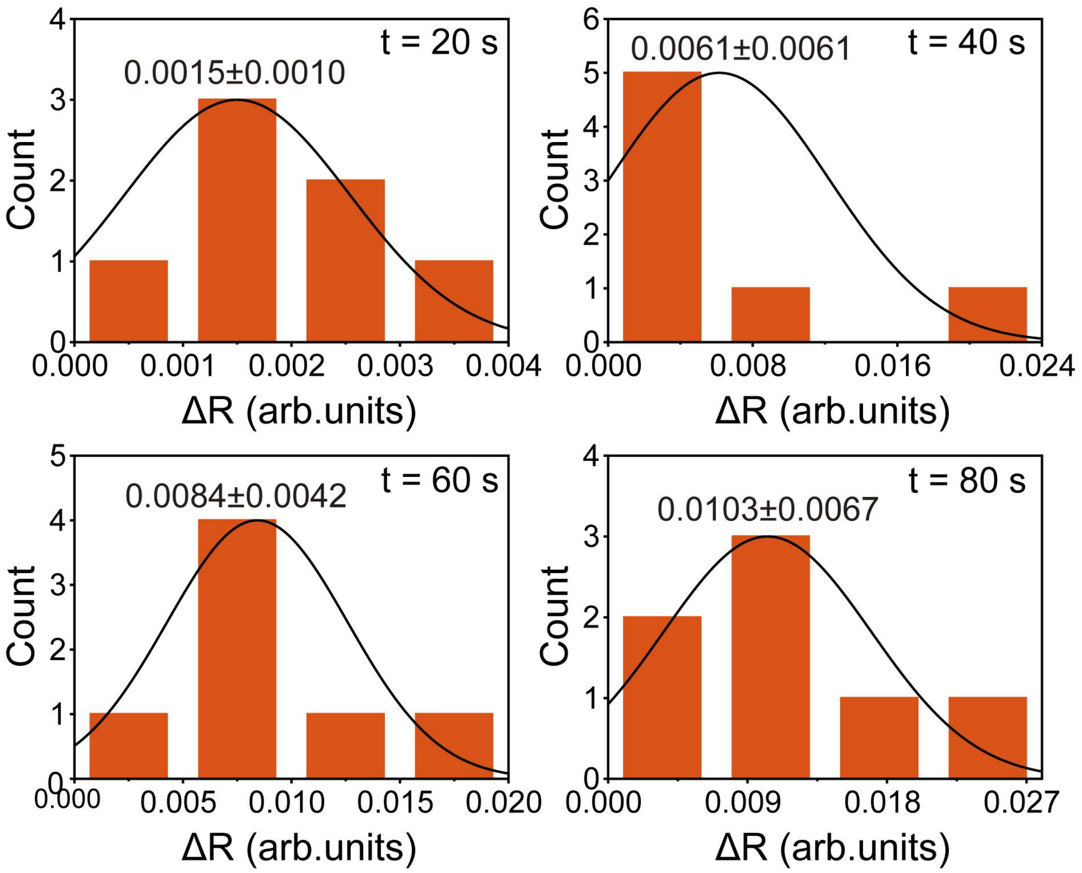

Supplementary Figure 27. Histogram distributions of COF-300-AR crystals in the presence of THF (0.88 bar) under different reaction time.

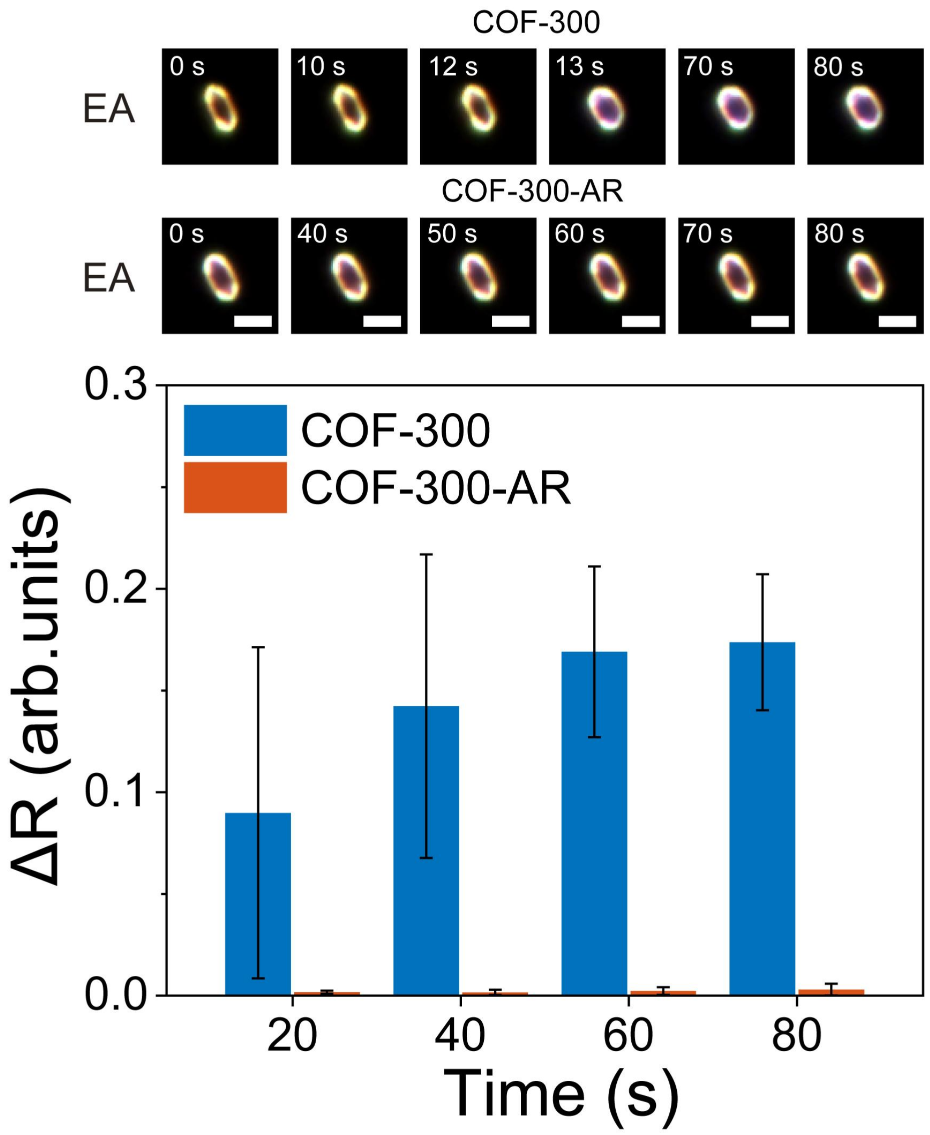

Supplementary Figure 28. Time-lapsed DFM images of single COF-300 and COF-300-AR in the presence of EA (0.76 bar) and the average roundness change (△R) of COF-300 and COF-300-AR crystals under different reaction time. Scale bars are 4 μm. Error bars stand for the standard deviations of △R.

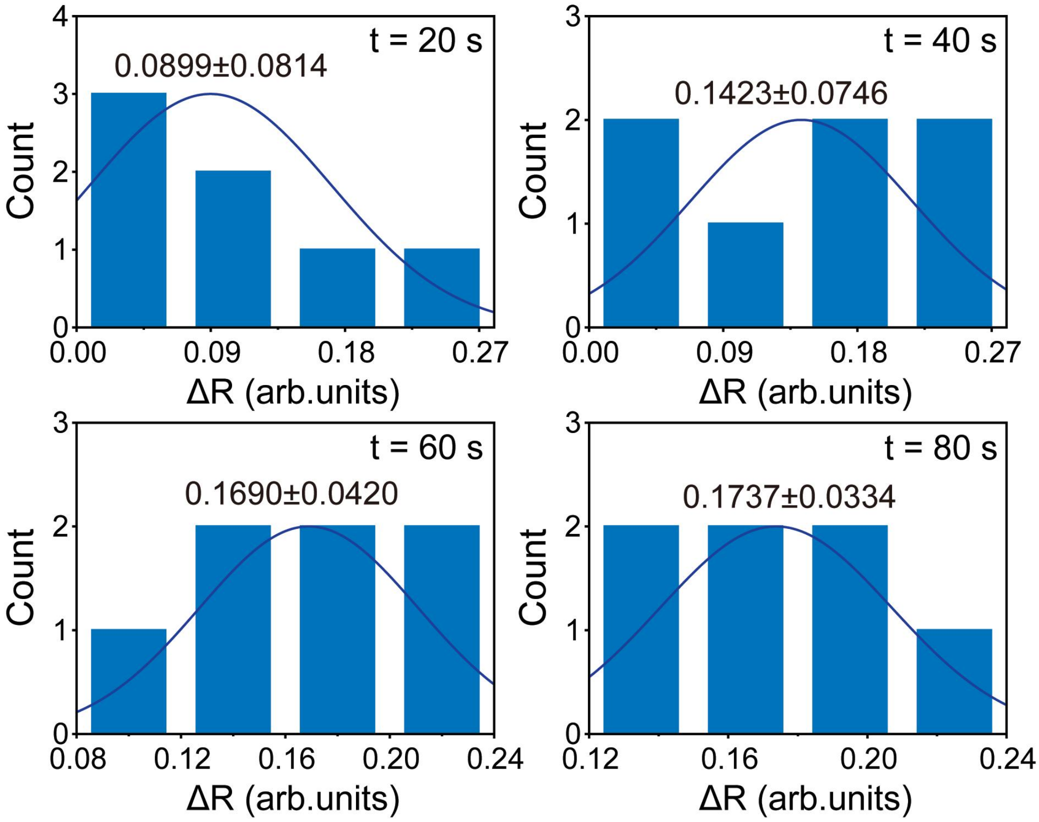

Supplementary Figure 29. Histogram distributions of COF-300 crystals in the presence of EA (0.76 bar) under different reaction time.

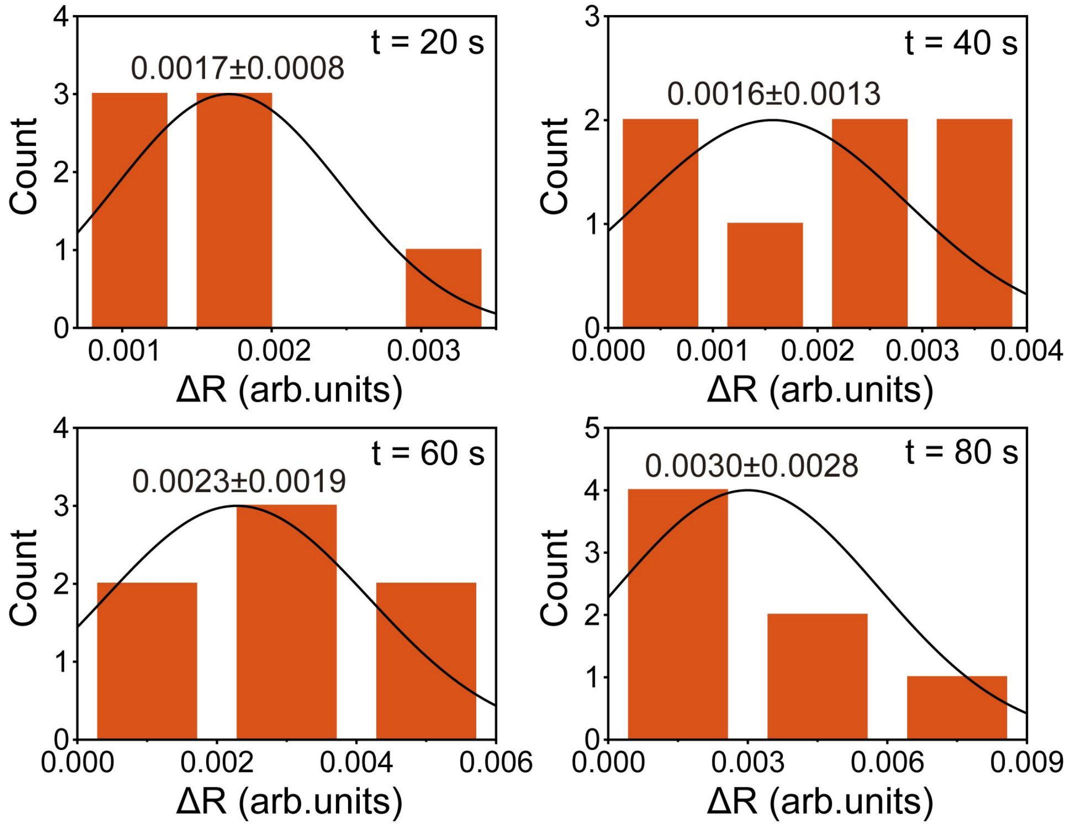

Supplementary Figure 30. Histogram distributions of COF-300-AR crystals in the presence of EA (0.76 bar) under different reaction time.

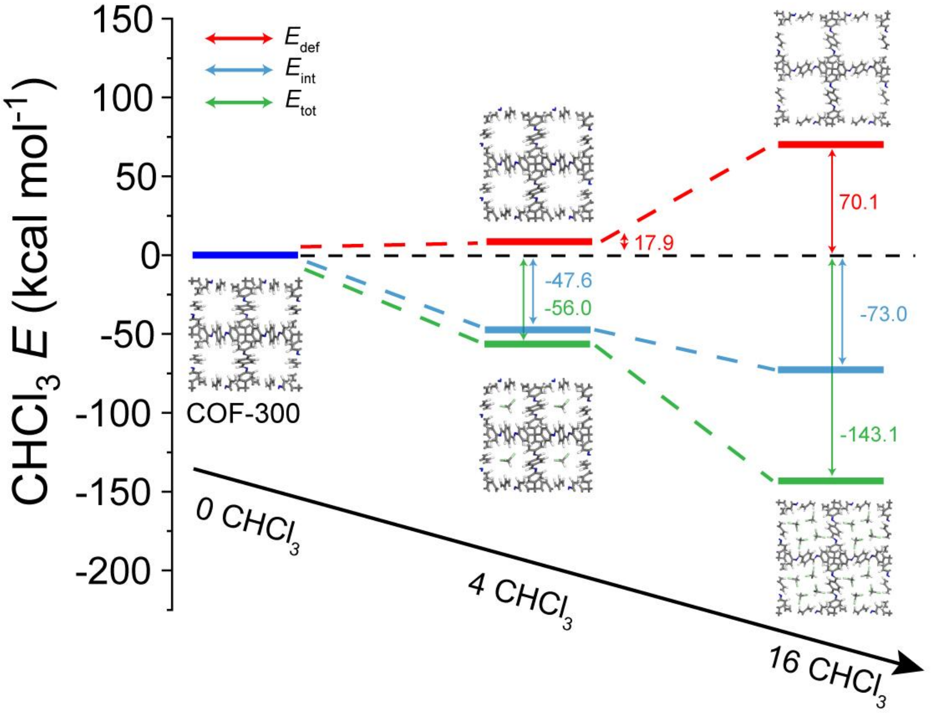

Supplementary Figure 31. Calculation of E_{def}, E_{int}, and E_{tot} during the sorption of different numbers of $CHCl_3$.

Based on the first-principles density functional theory and Monte Carlo simulations, a 'gate-opening' model for interpreting the guest-induced pore expansion is fabricated, which is adopted the model proposed by Kitagawa et al[1]. The deformation energy (E_{def}) and interaction energy (E_{int}) stand for the energy difference between closed- and open-pore states as well as the energy decrease after the sorption of $CHCl_3$. The sum of E_{def} and E_{int} is the total energy (E_{tot}). As displayed in Supplementary Figure 31, the E_{def} is about 17.9 kcal mol^{-1} for the sorption of four $CHCl_3$, and it varies to 70.1 kcal mol^{-1} after adsorbing sixteen $CHCl_3$, clarifying the requirement of the energy barrier for triggering the pedal motion.

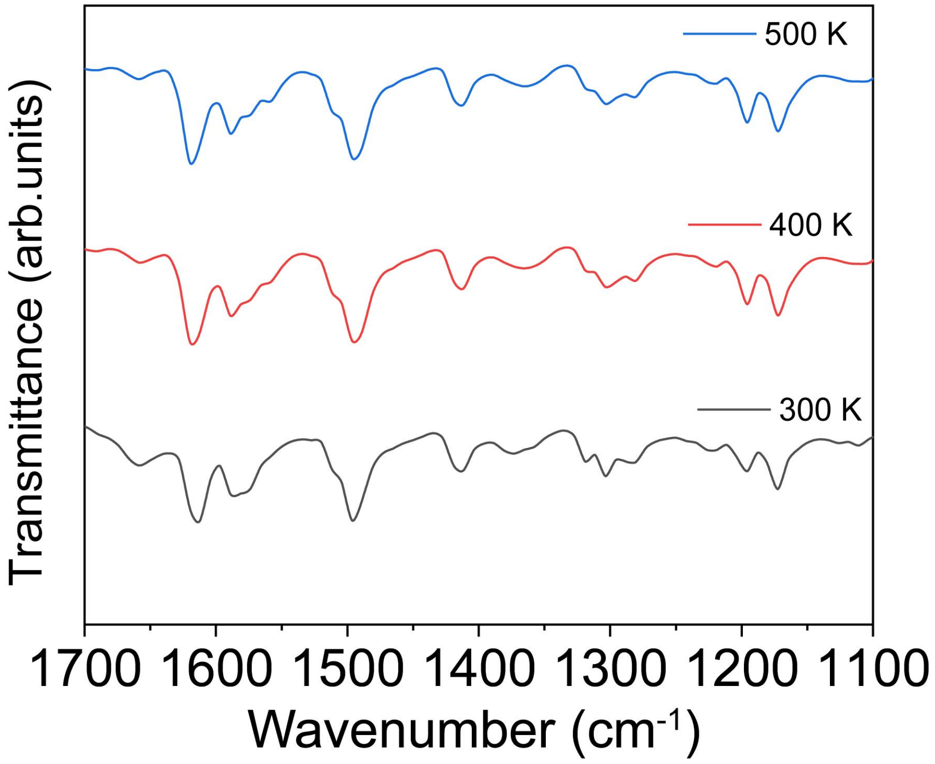

Supplementary Figure 32. Variable-temperature IR spectra of COF-300 under N_2 atmosphere.

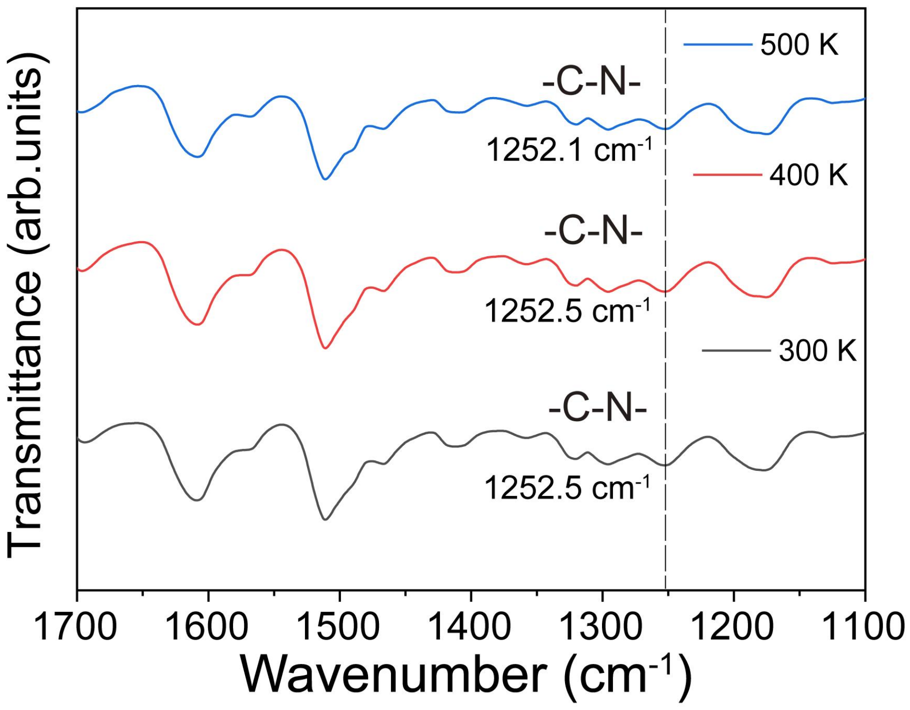

Supplementary Figure 33. Variable-temperature IR spectra of COF-300-AR under N_2 atmosphere.

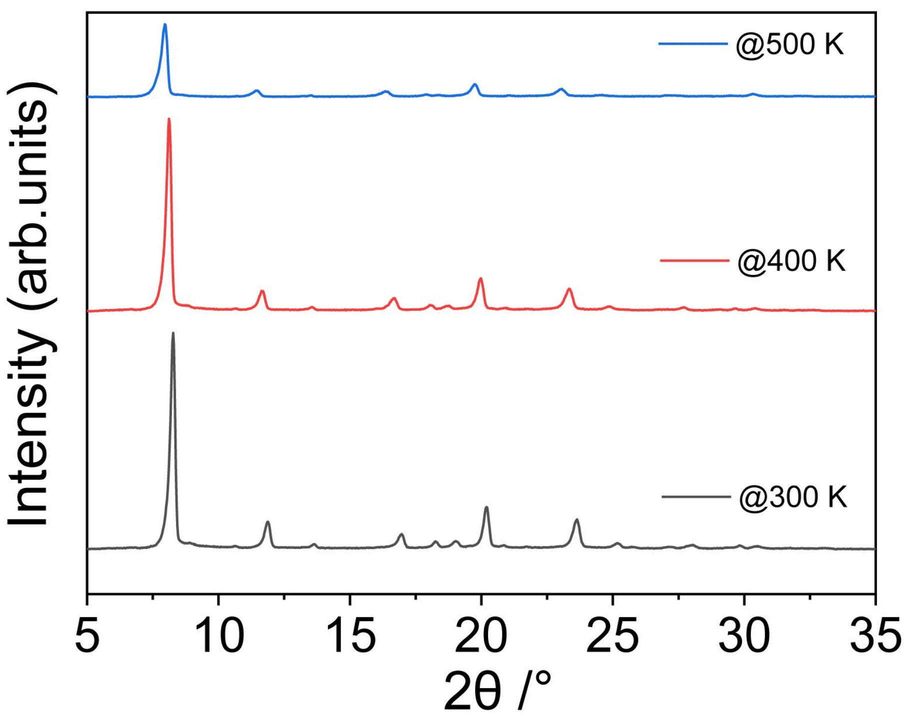

Supplementary Figure 34. Variable-temperature XRD patterns of COF-300 under N_2 atmosphere.

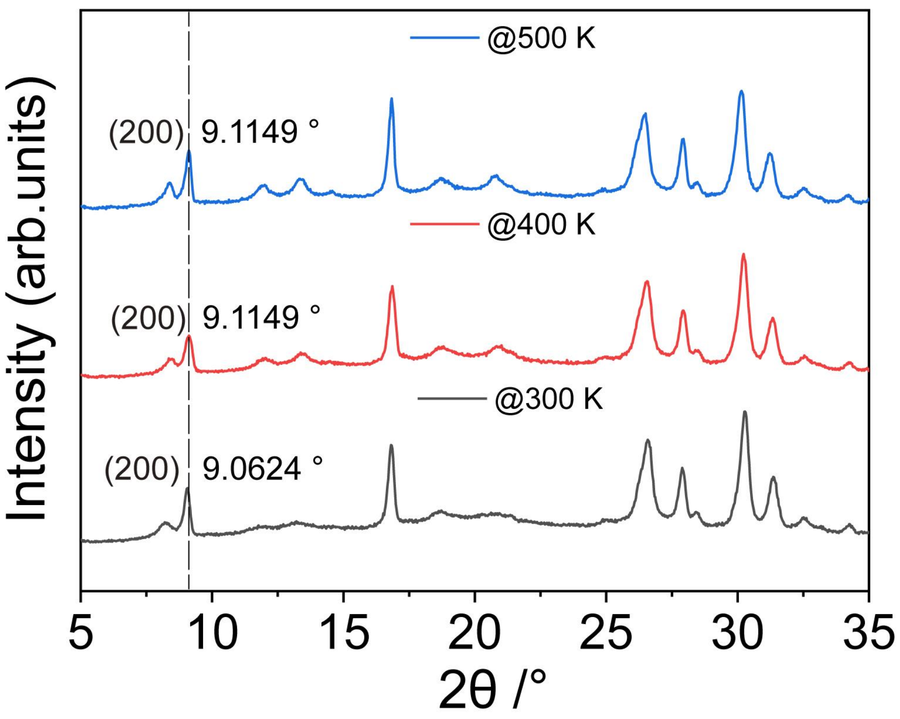

Supplementary Figure 35. Variable-temperature XRD patterns of COF-300-AR under N_2 atmosphere.

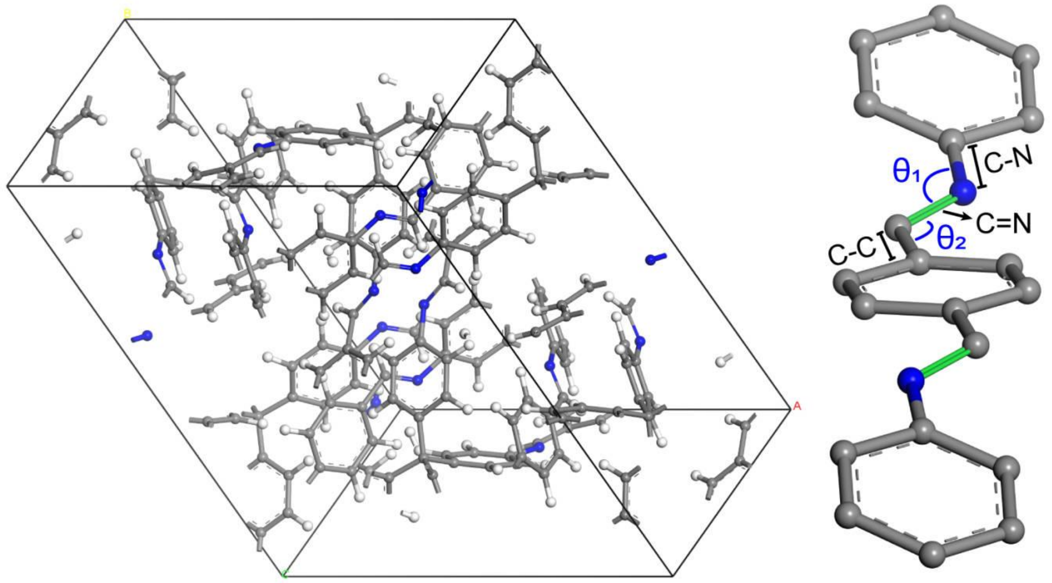

Supplementary Figure 36. The simulated configurations structure of COF-300 crystal and imine-linked configurations at 300 K.

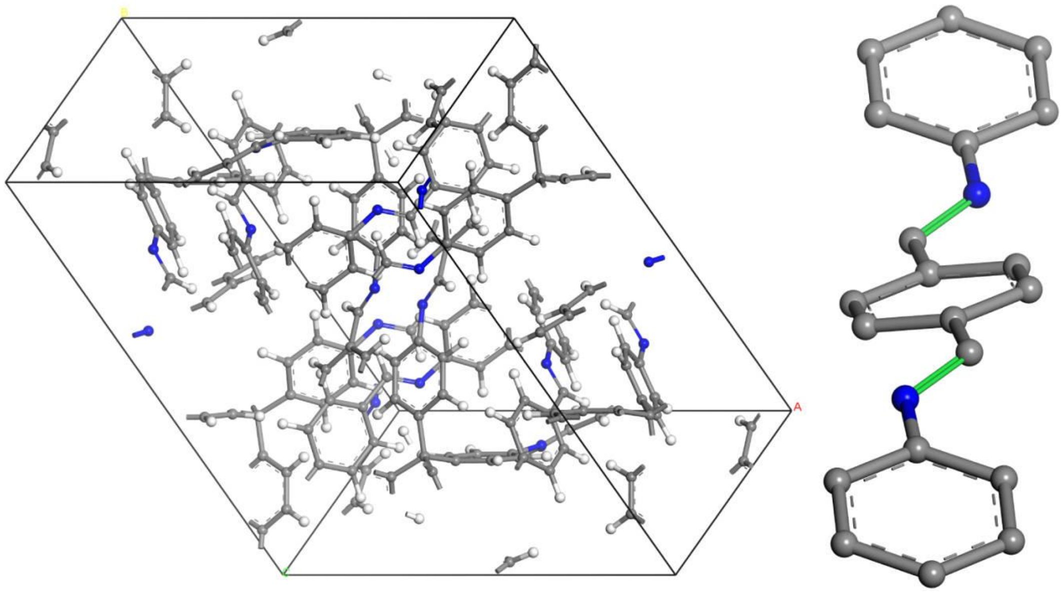

Supplementary Figure 37. The simulated configurations structure of COF-300 crystal and imine-linked configurations at 400 K.

.

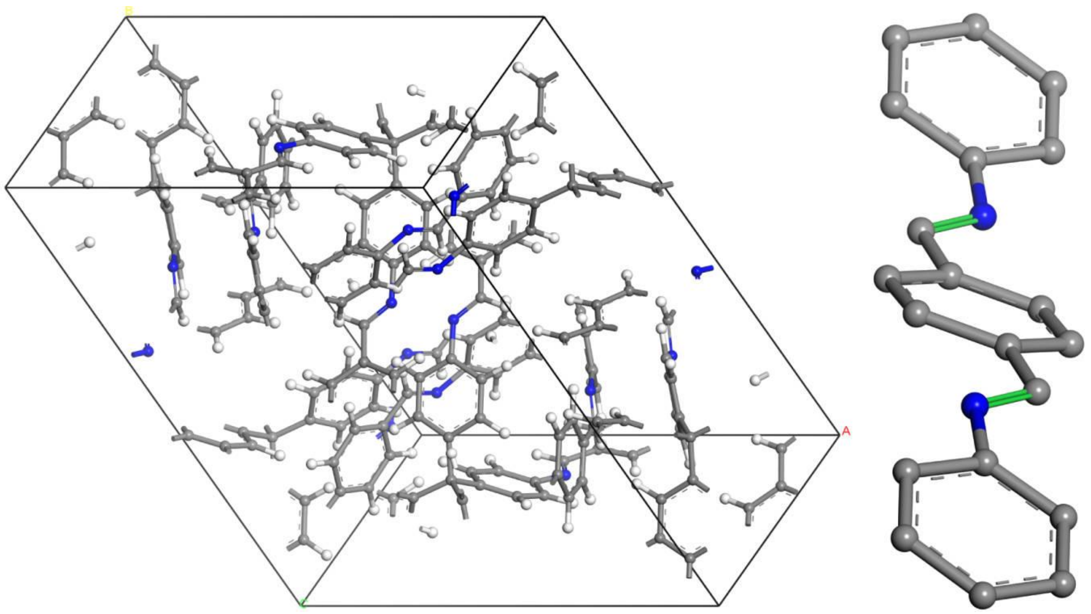

Supplementary Figure 38. The simulated configurations structure of COF-300 crystal and imine-linked configurations at 500 K.

Supplementary Table 1. Selected bond lengths in COF-300 crystal at different temperatures

COF	***Temperature / K***	***Covalent bond***	***Bond length / Å***
COF-300	300	C-N	1.423
		C=N	1.292
		C-C	1.456
	400	C-N	1.424
		C=N	1.293
		C-C	1.457
	500	C-N	1.422
		C=N	1.292
		C-C	1.457

Supplementary Table 2. Selected bond angles in COF-300 crystal at different temperatures

COF	*Temperature / K*	*Types*	*Bond angle / °*
COF-300	300	θ_1	125.946
		θ_2	119.091
	400	θ_1	125.472
		θ_2	119.303
	500	θ_1	127.040
		θ_2	118.663

Supplementary reference

1 Sen, S., Hosono, N., Zheng, J. J., Kusaka, S., Matsuda, R., Sakaki, S., & Kitagawa, S. Cooperative bond scission in a soft porous crystal enables discriminatory gate opening for ethylene over ethane. *J. Am. Chem. Soc.* **139**, 18313-18321 (2017).

2 Kang, C., Zhang, Z., Kusaka, S., Negita, K., Usadi, A. K., Calabro, D. C., L. S., Wang, Y. X., Zou, X. D., Huang, Z. H., Matsuda, R., &Zhao, D. Covalent organic framework atropisomers with multiple gas-triggered structural flexibilities. *Nat. Mater.* **22**, 636-643 (2023).

3 Zhou, Z. B., Han, X. H., Qi, Q. Y., Gan, S. X., Ma, D. L., & Zhao, X. A Facile, Efficient, and general synthetic method to amide-linked covalent organic frameworks. *J. Am. Chem. Soc.* **144**, 1138-1143 (2022).

4 Ma, T., Wei, L., Liang, L., Yin, S., Xu, L., Niu, J., Xue, H., Wang, X., Sun, J., Zhang, Y., &Wang, W. Diverse crystal size effects in covalent organic frameworks. *Nat. Commun.*, **11**, 6128 (2020).
