# Supplementary material for: Direct visual observation of pedal motion-dependent flexibility of single covalent organic frameworks
Source: Nat Commun. 2023 Aug 21;14:5061. doi: 10.1038/s41467-023-40831-8 (PMC10442449; doi:10.1038/s41467-023-40831-8)
Supplement: Supplementary file 3 — Description of Additional Supplementary Files [file 41467_2023_40831_MOESM3_ESM.pdf]

Description of Additional Supplementary Files

Title: Supplementary Movie 1

Description: DFM imaging of flexible deformation of single COF-300 crystal upon exposure to chloroform vapour.

Title: Supplementary Movie 2

Description: DFM imaging of single COF-300-AR particle in the presence of chloroform vapour.
